# Complete mitochondrial genome of the Florida manatee (*Trichechus manatus latirotris*, Sirenia)

**DOI:** 10.1590/1678-4685-GMB-2019-0210

**Published:** 2020-02-03

**Authors:** Sibelle T. Vilaça, Fabricio R. Santos

**Affiliations:** 1 Trent University, Environmental and Life Sciences Graduate Program, Peterborough, Ontario, Canada.; 2 Universidade Federal de Minas Gerais, Departamento de Genética, Ecologia e Evolução, Laboratório de Biodiversidade e Evolução Molecular, Belo Horizonte, MG, Brasil.

**Keywords:** Trichechus manatus, Florida manatee, Amazonian manatee, mitochondrial genome

## Abstract

The Florida manatee (*Trichechus manatus latirostris*) is an endangered subspecies of the West Indian manatee (*T. manatus*), which inhabits inland and marine waters of southeastern United States. In this study, we assembled the mitochondrial genome (mtDNA) of the Florida manatee from whole genome shotgun reads. As a result, we show that the currently annotated *T. manatus* mtDNA belongs to a different species, the Amazonian manatee (*T. inunguis*). The newly assembled Florida manatee mtDNA is 16,881 bp in length, with 13 protein-coding genes, two ribosomal RNAs (rRNAs), 22 transfer RNAs (tRNAs) and one non-coding control region (D-loop). Phylogenetic analysis based on the control region indicates the newly assembled mtDNA is haplotype A01, characteristic of *T. m. latirostris*, while the current mtDNA associated with the Florida manatee genome assembly has a Ti02 haplotype that is found in Amazonian manatees and hybrids.

The West Indian manatee (*Trichechus manatus*) is an aquatic mammal that belongs to the order Sirenia. West Indian manatees are taxonomically subdivided in two subspecies threatened with extinction: the Florida manatee (*T. m. latirostris*) that inhabits the southeastern United States, and the Antillean manatee (*T. m. manatus*) that is found in other coastal regions of the Americas (*T. manatus*) (Deutsch *et al.*, 2008)*.* The Amazonian manatee (*Trichechus inunguis*) is the second species found in South America, and it is highly specialized to freshwater environments of the Amazon River basin (Rosas, 1994). An interspecific hybrid population between West Indian and Amazonian manatees was recently characterized along the Guianas coastline and Amazon River mouth by our research team (Vilaça *et al.*, 2019; Lima *et al.*, 2019), showing introgressed individuals bearing mostly the Amazonian manatee mitochondrial DNA (mtDNA), and nuclear DNA from both parental species.

Here, we show that the current mitochondrial genome (GenBank accession NC_010302.1) associated with the Florida manatee genome (GenBank assembly accession GCA_000243295.1) and sequenced by Arnason *et al.* (2008), is actually related to the Amazonian manatee species and likely derived from an individual with hybrid ancestry. We assembled a new mitochondrial genome from whole genome shotgun sequences of a *T. m. latirostris* (Foote *et al.*, 2015). The complete Florida manatee mtDNA was deposited in GenBank under the accession number MN105083.

A total of 3,841,044,105 paired-end reads sequenced from a Florida manatee were retrieved from Genbank (SRS213934). To extract the shotgun reads belonging to the mtDNA, all raw reads were mapped to the published manatee mitochondrial genome (Arnason *et al.*, 2008, AM904728 or NC_010302.1). Mapped reads were extracted and an initial assembly was performed in Spades v3.12.0 (Bankevich *et al.*, 2012) using the published manatee mtDNA genome as a “trusted-contig”. To correct errors in the assembly and any biases caused by the reference-guided assembly, all reads were further mapped to the scaffolds obtained from the assembly using Geneious (Kearse *et al.*, 2012). The final consensus was annotated using MITOS (Bernt *et al.*, 2013) and Geneious, and visualized with Geneious. A phylogenetic tree using control region (D-loop) sequences was generated to confirm the mtDNA assignment of our new assembly to Florida manatee, since this is the only marker with population-level data for all species of manatees (Vianna *et al.*, 2006). A Bayesian tree was constructed using MrBayes v3.2.6 implemented in Geneious. Analysis consisted of two simultaneous runs with four Markov chains using 1,100,000 generations, with a burn-in of 10% of the initial trees and sampling every 200 generations. The GTR+G was used as the nucleotide substitution model.

A total of 1,383,966 reads mapped to the reference mtDNA sequence (Arnason *et al.*, 2008). The complete Florida manatee mitochondrial genome is 16,881 bp in length, and the assembly had an average coverage of 4,242 X. Similar to other vertebrate mitochondrial genomes, it has 37 genes, divided in 13 protein coding genes, 22 tRNA genes, two rRNA genes (12S rRNA and 16S rRNA), and one control region (Figure 1). Comparing the new assembly (GenBank accession MN105083) to the reference mtDNA sequence (GenBank accession AM904728), a total of 107 polymorphisms were observed, including 104 single nucleotide changes (SNPs) and three insertions/deletions (indels). Twenty one out of 104 SNPs represented non-synonymous substitutions.

**Figure 1:**
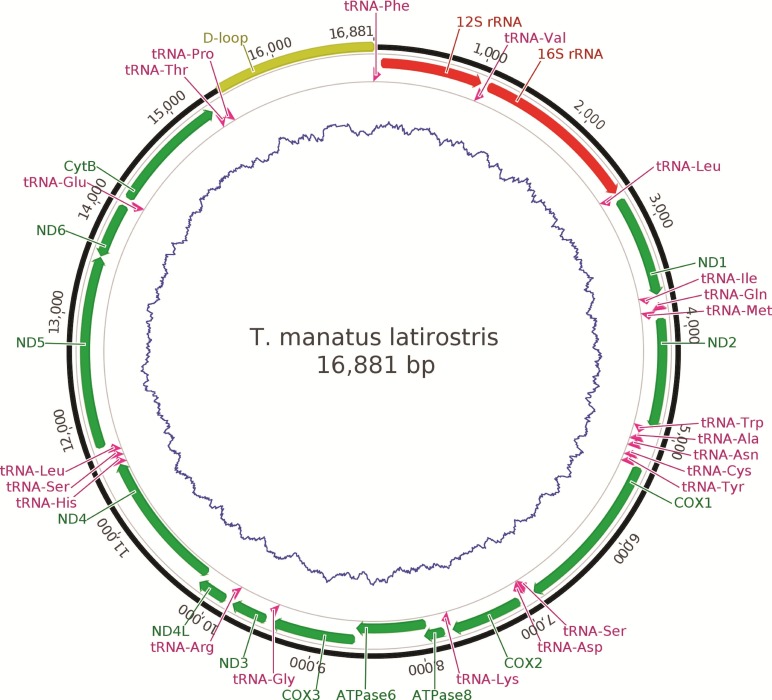
Schematic representation of the *Trichechus manatus latirostris* mitogenome depicting the annotated regions. The inner circle (blue) represents the GC content.

The Bayesian phylogeny reached convergence (ESS > 400), and the phylogenetic tree recovered similar relationships between clades as in previous studies (Figure 2) (Vianna *et al.*, 2006). The mitogenome sequenced by Arnason *et al.* (2008) grouped with other Amazonian manatees, while our newly assembled mitogenome grouped as expected within *T. manatus* cluster I (as defined by Vianna *et al.*, 2006), found in Florida/USA, Mexico, Colombia, Venezuela, Central America, and Antilles. The control region haplotype from the newly assembled genome was identical to A01, the most common mtDNA haplotype found in Florida, USA. On the other hand, the mtDNA haplotype currently associated to the reference Florida manatee genome was T02, a *T. inunguis* mtDNA haplotype found in French Guiana and reported in hybrid manatees (Vianna *et al.*, 2006; Santos *et al.*, 2016).

**Figure 2:**
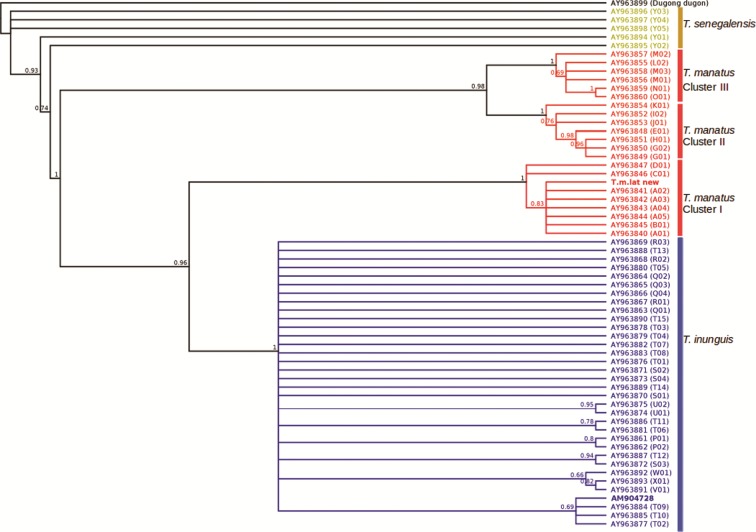
Bayesian phylogenetic tree from mtDNA control region sequences showing the phylogenetic position of the newly assembled mtDNA genome (*T.m.lat* new) and the current *T. manatus* reference mtDNA genome (AM904728) in bold. Clusters are shown as defined by Vianna *et al.* (2006).

Here we demonstrate the importance of correct species assignment in genomic resources. The previous West Indian (*T. manatus*) manatee mitogenome was sampled from a supposed Antillean manatee (Arnason *et al.*, 2008), and given its sequence similarity to the Amazonian manatee, this sample is a likely descendent of hybrids known to occur in the mouth of the Amazon River and along the Guianas coastline (Santos *et al.*, 2016; Vilaça *et al.*, 2019). A correct reference is specifically important in the case of an endangered species or subspecies, as inaccurate conclusions regarding sharing of haplotypes between species, based on the wrong reference might occur, as shown by Bonvicino *et al.* (2019), leading to inappropriate interpretations and conservation strategies. Our newly assembled mitogenome provides a correct reference for Florida (and West Indian) manatees for future studies.
